# Can Parents Improve the Quality of Life of Their Children with Attention Deficit Hyperactivity Disorder?

**Published:** 2019-04

**Authors:** Maryam Kousha, Mohammad Abbasi Kakrodi

**Affiliations:** 1 Kavosh Cognitive Behavior Sciences and Addiction Research Center, Department of Child and Adolescent Psychiatry, School of Medicine, Guilan University of Medical Sciences, Rasht, Iran.; 2 Kavosh Cognitive Behavior Sciences and Addiction Research Center, Department of Psychiatry, School of Medicine, Guilan University of Medical Sciences, Rasht, Iran.

**Keywords:** *Attention Deficit Hyperactivity Disorder*, *Children*, *Pediatric Quality of Life*

## Abstract

**Objective:** The aim of this study was to evaluate the effectiveness of mothers’ Group psychoeducation on Quality of Life (QoL) of children with Attention Deficit Hyperactivity Disorder.

**Method** **:** In this clinical trial, 60 mothers of ADHD children were randomly divided into two groups (30 participants in each group). An educational program based on Positive Parenting Program (Triple P) was performed for the intervention group, while only pharmacotherapy was provided for the control group. Pediatric Quality of Life Inventory (Peds QL) was completed by all 60 mothers before, eight week, and three months after intervention. Data were analyzed using mean and standard deviation, and K-square or paired t test were used for data analysis.

**Results: **A total of 60 mothers participated in this study. Of their children, 80% were boys and 20% were girls. The mean of the total score of QoL increased significantly in the intervention group at week eight and three months after the intervention. Also, the mean scores of emotional, social, school and psychosocial domains, but not physical domain of QoL, found to be higher in ADHD children after intervention (p< 0.05).

The total score of QoL and mean scores of domains increased in the posttest in the control group, but it was not significant (p> 0.05).

**Conclusion: **A significant increase in the total score of QoL was reported by mothers in the posttest compared to the pretest in the experimental group, which showed that educating parents can improve the QoL of their ADHD children.

Attention deficit hyperactivity disorder (ADHD) is the most common neurodevelopmental disorder in children and adolescents (1). ADHD is characterized by a pattern of diminished sustain attention and increased impulsivity or hyperactivity that affects 5% to 8% of school-aged children with 60% to 85% continuing to adolescence and up to 60% continuing to adulthood (2). ADHD symptoms affect all aspects of life (2, 3 and 4). Those with ADHD have serious impairment in cognitive, emotional, social, interpersonal, and academic performance in life (2, 3, 4). ADHD is frequently associated with comorbid conditions, including learning disability (LD), oppositional defiant disorder (ODD), conduct disorder (CD), substances use disorders (SUD), and depression and anxiety disorders (2, 4). 

ADHD increases low self-esteem, poor academic function, family and peer relationships problems, and disruptive behaviors in children and adolescent (4).

Considering the pervasive symptoms of ADHD, children with diagnosis of ADHD, experience impaired well-being or quality of life compared to healthy children (4).

According to the definition of World Health Organization (WHO), one of the comprehensive assessments for youths with ADHD based on the multidimensional view of health is health- related quality of life (HRQOL) (3, 4). HRQOL is a personal subjective perception of the impact of health conditions (e.g., disease and treatment) on physical, psychological, or social well-being (3).

The results of a meta-analysis of quality of life in children with ADHD indicated that ADHD impact a child or adolescent HRQOL negativity, with moderate effect in physical and severe effect in psychosocial domains, including emotional and social aspects and school performance (3). Danckaerts et al, in their review, concluded that the effect of ADHD on QoL is significant. They found that parents of ADHD children estimate negative emotions more than children themselves (5). The only study of QoL on Iranian ADHD children showed that Pediatric Quality of Life Inventory (Peds QL) has adequate validity and reliability; this study also obtained lower scores of QoL in ADHD children than control group (6). Low QoL is an essential problem for ADHD patients (7) and can predict adverse psychosocial outcomes for them (8). Thus, QoL may be a good predictor of treatment efficacy.

Many studies concluded that pharmacological treatments are effective for managing the ADHD core symptoms (2). In recent years, there has been increased attention on psychosocial interventions in ADHD. Parent training programmers provide psychosocial interventions to enable parents to manage their children’s challenging behaviors (9). The results of a review in 2011 showed positive effects of parent training on behavior of children with ADHD (9). Another systematic review in 2015 found a moderate reduction in ADHD symptoms (10). Several studies showed the impact of medication treatment on QoL of ADHD patients, but few studies have investigated the effectiveness of psychosocial treatment on QoL of these patients (5).

Studies examined the effectiveness of triple p on Iranian mothers of ADHD children and showed significant improvement in parenting style, mother-child relationship, and lower rates of child misbehavior reported by mothers (11, 12). 

Qol is an important measure of outcome in children’s mental health (5) and the most important goal of treatment efficacy (13). In addition, psychiatric medications appear to be inadequate for optimal outcome of ADHD (1). 

Considering the effectiveness of non-drug treatment and its good compliance as reported by the parents of children with ADHD as well as the importance of mother-child interaction in enhancing the QoL of these children, this study was conducted to assess the effectiveness of mother’s educational group therapy in enhancing parenting skills on Peds QL of ADHD children.

## Materials and Methods

This was a semi-experimental (pretest – posttest) with a control group. The study was approved by the Ethical Committee at Research Center of Guilan University of Medical Sciences and in accordance with Declaration of Helsinki (IRCT ID: IRCT201701297237N2).

***Participants and Procedure***

Participants were recruited from mothers of children and adolescents who had the diagnosis of ADHD based on a diagnostic interview by a child and adolescent psychiatrist according to Diagnostic and Statistical Manual Disorders, Fifth Edition (DSM – 5) criteria in an outpatient child and adolescent psychiatric clinic in Rasht, a city in the north of Iran in 2015-2016. 

Sample consisted of 60 mothers of 8-12 year-old ADHD children who were on drug treatment with methylphenidate, with therapeutic and effective dose started at least 2 months prior to the study. Mothers of ADHD children without any comorbidity were included in this study to eliminate the effects of other psychiatric disorders on parenting skills. Eligible participants were mothers who applied for educational parenting group. They did not have any psychiatric or active medical disorder based on a diagnostic interview by a child and adolescent psychiatrist according to Diagnostic and Statistical Manual Disorders, Fifth Edition (DSM – 5) criteria. After providing complete explanation and obtaining written informed consent, pretests were administered for all of mothers, then, they were randomly divided into experimental and control groups. Posttests were performed for experimental and control mothers after 8 sessions of educational parenting program and 3 months later. 

The control group were mothers in a waiting list whose ADHD children received the usual treatment (methylphenidate with therapeutic and effective dose) and were monthly visited by a child and adolescence psychiatrist. After data collection, the program was also performed for the control group. The educational program was a performed by a child and adolescent psychiatrist and a psychiatry resident as a co-therapist.

***Intervention***

The educational program used in this study was based on Positive Parenting Program (Triple P), conducted in groups of 10-15 parents. The program consists of 8 two-hour sessions with the opportunity for parents to learn by observation, discussion, practice, and feedback (12, 14). The contents of the training sessions are listed in Table 1.

***Measures***

Pediatric Quality of Life Inventory (Peds QL) is a 23-item questionnaire for 8-12-year-old children and include child report and parent report. In this study, parent report form was used to assess the QoL of ADHD children. The Peds QL measures HRQOL with physical functioning (5 items), emotional functioning (5 items), social functioning (5 items), and school functioning (5 items) using a 5-point scale (0 = never, 1 = almost never, 2 = sometimes, 3 = often, 4 = always). Then, these items are reserved to scores 0 to 100, with higher scores indicating better QoL (1 5). The psychometric properties of the Peds QL (reliability, validity, and sensitivity to change) for studies conducted in countries whose native language is not English is acceptable (3). The reliability and validity of this questionnaire were tested in Iran and found to be adequate (alpha = 0.71-0.89) (6). 

This instrument is a comprehensive questionnaire that includes various areas of a child's performance and evaluates different areas of his/ her physical, emotional, social, and school performance (3). 

***Statistical Analysis***

Data were analyzed using SPSS software version 22. Also, mean and standard deviation were used for descriptive statics, and K-square or paired t test used for data analysis.

**Table1:** The Contents of the Training Sessions

**Session**	**Items**
1	Reasoning, statement of goals and rules of the group, explaining the symptoms of ADHD by mothers, Definition, prevalence, etiology and general principles of treatment, correcting parents' misconceptions about the nature of the disease
2	Expression of child-parent interactions and general principles of behavioral shaping
3	Group discussion about the previous session, training regarding general procedures and conditions reinforce the behavior, encouraging and its variants
4	Group discussion about the previous session, the teaching token economy, homework for practicing this at home
5	Group discussion about the previous session, teaching new skills and managing misbehavior
6	Group discussion about the previous session and review of the assignments related to the past session, how to reduce the child's inappropriate behavior, planned ignoring, logical consequences
7	Group discussion about the previous session and the review of assignments related to the past session, identifying children's problem-solving, behaviors in relation to the school
8	Group discussion about the previous session and the study of the related assignments, developing coping plans for high-risk situations, closing the program

**Table2:** Sociodemographic Characteristics of Mothers and Their Children

	**intervention**		**Comparison**		**P**
	**mean**	**SD**	**mean**	**SD**	
Age of child (year)	9.30	1.3	9.43	1.4	0.5
Age of mother (year) 85.33 2.34	36.00	2.58	35.33	2.34	0.299
Gender of child		83.3			
Male	25	%3	23	76.7	
Female	5	%16.7	7	23.3	0.7
Education of mother					
≤ High school	10	33.3	10	33.3	
≥ Diploma	17	56.7	11	36.7	0.117
≥ Licentiate	3	10	9	30	
Family income (Rials)					
0-15.000.000	21	70	16	53.3	
15.000.000-25.000.000	5	16.7	9	30.00	0.381
25.000.000-50.000.000	4	13.3	5	167	

**Table3:** Mean Peds QL Domain and Total Scores in Mothers of ADHD Children before Intervention, 8 Weeks and 3 Months after Intervention

	**Before ** **Intervention**		**8 Weeks After ** **Intervention**		**P value** †	**3 Months After ** **Intervention**		**P value** ††
	**mean**	**SD**	**mean**	**SD**				
Physical Functioning	63.494	23.943	69.602	21.220	0.079	74.375	20.990	0.087
Emotional Functioning	49.629	25.529	62.592	20.492	0.003	58.809	22.355	0.077
Social Functioning	60.384	19.179	69.615	21.303	0.017	68.750	21.574	0.077
School Functioning	58.888	18.310	66.296	19.738	0.041	64.000	20.365	0.145
Psychosocial Functioning	55.897	16.160	66.089	16.158	0.001	62.368	18.124	0.026
Total score	57.165	15.340	67.098	14.177	0.002	65.425	16.026	0.033

† Difference between before intervention and 8 weeks after intervention

†† Difference between before intervention and 3 months after intervention

**Figure1:**
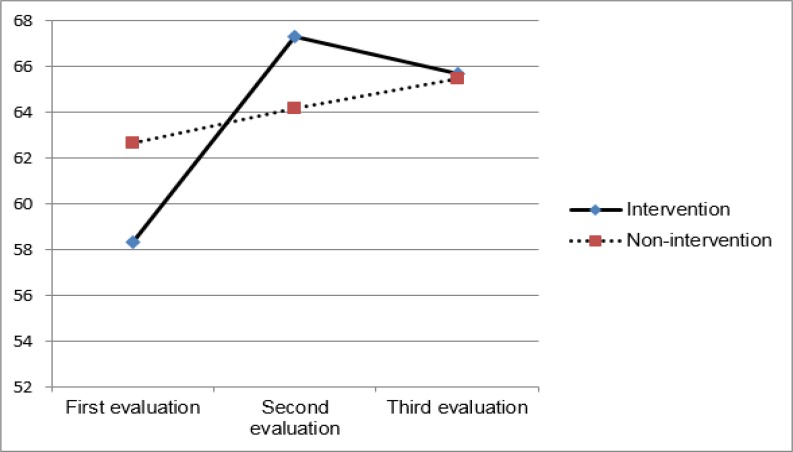
Difference between the Experimental and Control Groups in the Scores of QOL during Study

## Results

In this study, 70 mothers were assessed for participation, and of their children, 80% were boys and 20% were girls. At the beginning of the study, 10 mothers were excluded because they did not fulfill the inclusion criteria or provide consent. Then, 60 mothers were randomly divided into 2 groups. From the 30 mothers in the experimental and control groups, 21 and 24 mothers completed the sessions, respectively . Table 2 presents the sociodemographic characteristics of mothers and children in the 2 groups. Mothers in the experimental and control groups were matched for educational level and family income, and no significant difference was found between the groups before intervention. 

The results revealed that the mean of total score of QoL increased significantly in the experimental group at week 8 and 3 months after intervention. Similarly, the mean scores of emotional, social, school, and psychosocial domains of quality of life were higher in ADHD children after intervention (Table 3).

According to Figure 1, there were no significant differences between the experimental and control groups in the scores of QoL in different areas and the total score at baseline. The scores were slightly higher in the control group, which might be due to a better economic situation or higher maternal education in this group. However, these 2 items, and other demographic items, did not differ significantly between the 2 groups (Table 2). 

There were no differences between the 2 groups in the mean scores for each item and the total QoL before intervention, at week 8 and 3 months after intervention (p> 0.05). 

Figure 1 demonstrates the compression of the total score of QoL in the 2 groups before the intervention, at week 8, and 3 months after intervention.

## Discussion

The aim of this study was to investigate the effectiveness of mother’s group psychoeducation on QoL of children with ADHD. To our knowledge, this was the first study to evaluate the relationship between a multidimensional and validated measure of health and non-pharmacological intervention for ADHD patients in Iran.

The most important finding of this study was a significant increase in the total score of QoL reported by parents in the posttest compared to the pretest in the experimental group, while this difference was not significant in the control group. Using effective parenting skills, better understanding of the disorder, and reducing the stress of parents through group discussion can lead to better parent-child interaction and may reduce punishment and improve QoL of children from the perspective of parents.

The increase in QoL scores in the intervention group was significant in all domains, except for the physical domain, which is justifiable due to the psychological nature of the disorder, and it is consistent with the results of Lee et al’s meta-analysis (3).

The mean of baseline QoL scores based on parents' report in the intervention and nonintervention group was 58 and 62, respectively, which is lower than the healthy population of 8-17-year-old youths without a history of this disorder or other problems, using the same quality of life assessment tool in Iran. In a study, the mean of the total score of parents reports of QoL was 77 (15). This finding confirms the lower QoL in ADHD youths compared to their healthy peers, and it is consistent with many studies in the world (5, 6, 16, 17). QoL is closely linked to the psychological and social functions, and ADHD affects all areas of the child's life, especially his or her social relationships at home, school with family, peers, and teachers.

According to the results of this study, the total scores of QoL in both intervention and not interventional groups were higher in the second and third follow-ups compared to the baseline. There was no significant difference between the 2 groups in improving the scores of QoL, but the difference between the 2 groups 3 months after intervention was less than 8 weeks after the intervention, and it seems that the effect of parenting education on mothers was initially more powerful (Figure 1). This can be explained by the fact that the effectiveness of educational intervention reduced after the sections (5).

No significant difference was found between the 2 groups after the intervention. Considering that drug therapy is the most important part of treatment for ADHD and that both interventional and non-interventional groups were under drug treatment with methylphenidate, this result confirmed the role of drug therapy in improving QoL in children with ADHD (18, 19).

Few studies have focused on the impact of non-pharmacological treatments on the QoL of children with ADHD. In the meta-analysis conducted in 2010 on the QoL of these children, there was only 1 study on the effect of psychological training on QoL, which indicated improvement in the QoL scores in the first 3 months after the onset of treatment and they emphasized the greater impact of drug therapies (5). Perhaps close contact with a psychiatrist in weekly sections had a positive effect on mothers’ attitude with their ADHD children during the active period of group therapy.

## Limitation

In this study, the severity of ADHD symptoms and subgroups of ADHD were not considered. Although the instrument used was comprehensive, it would have been better if we had used several tools.

## Conclusion

Significant increase was observed in the total score of QoL reported by mothers in the posttest compared to the pretest in the experimental group, indicating that providing education to mothers can improve the QoL of their ADHD children.
